# Crystal structure and solvent-dependent behaviours of 3-amino-1,6-diethyl-2,5,7-trimethyl-4,4-di­phenyl-3a,4a-di­aza-4-bora-*s*-indacene

**DOI:** 10.1107/S2056989017002213

**Published:** 2017-02-14

**Authors:** Lijing Yang, Brett Drew, Ravi Shekar Yalagala, Rameez Chaviwala, Razvan Simionescu, Alan J. Lough, Hongbin Yan

**Affiliations:** a Department of Chemistry, Brock University, 1812 Sir Isaac Brock Way, St Catharines, Ontario, L2S 3A1, Canada; b Department of Chemistry, University of Toronto, 80 St George Street, Toronto, Ontario, M5S 3H6, Canada

**Keywords:** crystal structure, BODIPY, excitation and emission, fluorescence, NMR spectroscopy, solvent dependence

## Abstract

In the title compound (3-amino-4,4-diphenyl-BODIPY), C_28_H_32_BN_3_, the central six-membered ring has a flattened sofa conformation, with one of the N atoms deviating by 0.142 (4) Å from the mean plane of the other five atoms, which have an r.m.s. deviation of 0.015 Å. The dihedral angle between the two essentially planar outer five-membered rings is 8.0 (2)°. In the crystal, mol­ecules are linked *via* weak N—H⋯π inter­actions, forming chains along [010]. The com­pound displays solvent-dependent behaviours in both NMR and fluorescence spectroscopy. In the ^1^H NMR spectra, the aliphatic resonance signals virtually coalesce in solvents such as chloro­form, di­chloro­methane and di­bromo­ethane; however, they are fully resolved in solvents such as dimethyl sulfoxide (DMSO), methanol and toluene. The excitation and fluorescence intensities in chloro­form decreased significantly over time, while in DMSO the decrease is not so profound. In toluene, the excitation and fluorescent intensities are not time-dependent. This behaviour is presumably attributed to the assembly of 3-amino-4,4-diphenyl-BODIPY in solution that leads to the formation of noncovalent structures, while in polar or aromatic solvents, the formation of these assemblies is disrupted, leading to resolution of signals in the NMR spectra.

## Abstract 2

3-Amino-1,6-diethyl-2,5,7-trimethyl-4,4-diphenyl-4-bora-3a,4a-di­aza-*s*-indacene displays solvent-dependent behaviour in both NMR and fluorescence spectroscopy.

## Chemical context   

4,4-Di­fluoro-3a,4a-di­aza-4-bora-*s*-indacene (BODIPY, see Scheme 1), as an attractive fluoro­phore, has found many applications in material sciences, as sensors and in labelling biomolecules such as proteins, lipids and nucleic acids (Ulrich *et al.*, 2008 ▸; Loudet & Burgess, 2007 ▸; Ziessel *et al.*, 2007 ▸; Tram *et al.*, 2011 ▸; Lu *et al.*, 2014 ▸; Bessette & Hanan, 2014 ▸). In our efforts to develop new BODIPY labelling chemistry, BODIPY analogues bearing an amino group, such as 3-amino-4,4-di­fluoro- and 3-amino-4,4-diphenyl-BODIPY, are being sought. While 3-amino-4,4-di­fluoro-BODIPY has been synthesized pre­viously (Liras *et al.*, 2007 ▸), a unique solvent-dependent behaviour of 3-amino-4,4-diphenyl-BODIPY, but not 3-amino-4,4-di­fluoro-BODIPY, was observed by NMR. In this regard, the resonance signals of the aliphatic protons are fully resolved in solvents such as DMSO-*d*_6_, but coalesced in solvents such as CDCl_3_. We herein report the solvent-dependent behaviour of 3-amino-4,4-diphenyl-BODIPY analogues as observed in the ^1^H NMR and in excitation and emission spectroscopy. The crystal structure suggests that the title compound could form noncolvalent assemblies in solvents such as CDCl_3_, leading to its solvent-dependent behaviours in NMR and fluorescence spectroscopy.

### Synthesis of BODIPY 2b   

**Figure 1:**
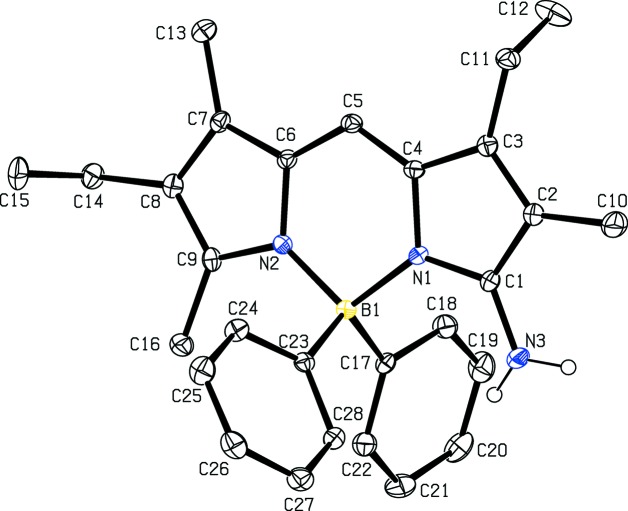
The mol­ecular structure of the title compound, with displacement ellipsoids drawn at the 30% probabilty level. H atoms are not shown.

The presence of an amino group in BODIPY allows for functional-group transformation and potential applications in labelling biomolecules. Towards the synthesis of amino BODIPY, an intriguing chemistry was recently described (Liras *et al.*, 2007 ▸). In this chemistry, a one-pot reaction of a substituted pyrrole in the presence of sodium nitrite, acetic acid and acetic anhydride, followed by treatment with boron trifluoride dietherate, led to the formation of a mixture of amino **2a** and acetimido BODIPY **3a** (see Scheme 2, *R* = F). Following this approach, 3-amino-1,6-diethyl-2,5,7-trimethyl-4,4-diphenyl-3a,4a-di­aza-4-bora-*s*-indacene (BODIPY **2b**, see Scheme 2 and Fig. 1 ▸) was synthesized in very low yield (typically <5%), where boron trifluoride diethyl etherate was replaced with di­phenyl­boron bromide (Scheme 2, *R* = Ph).
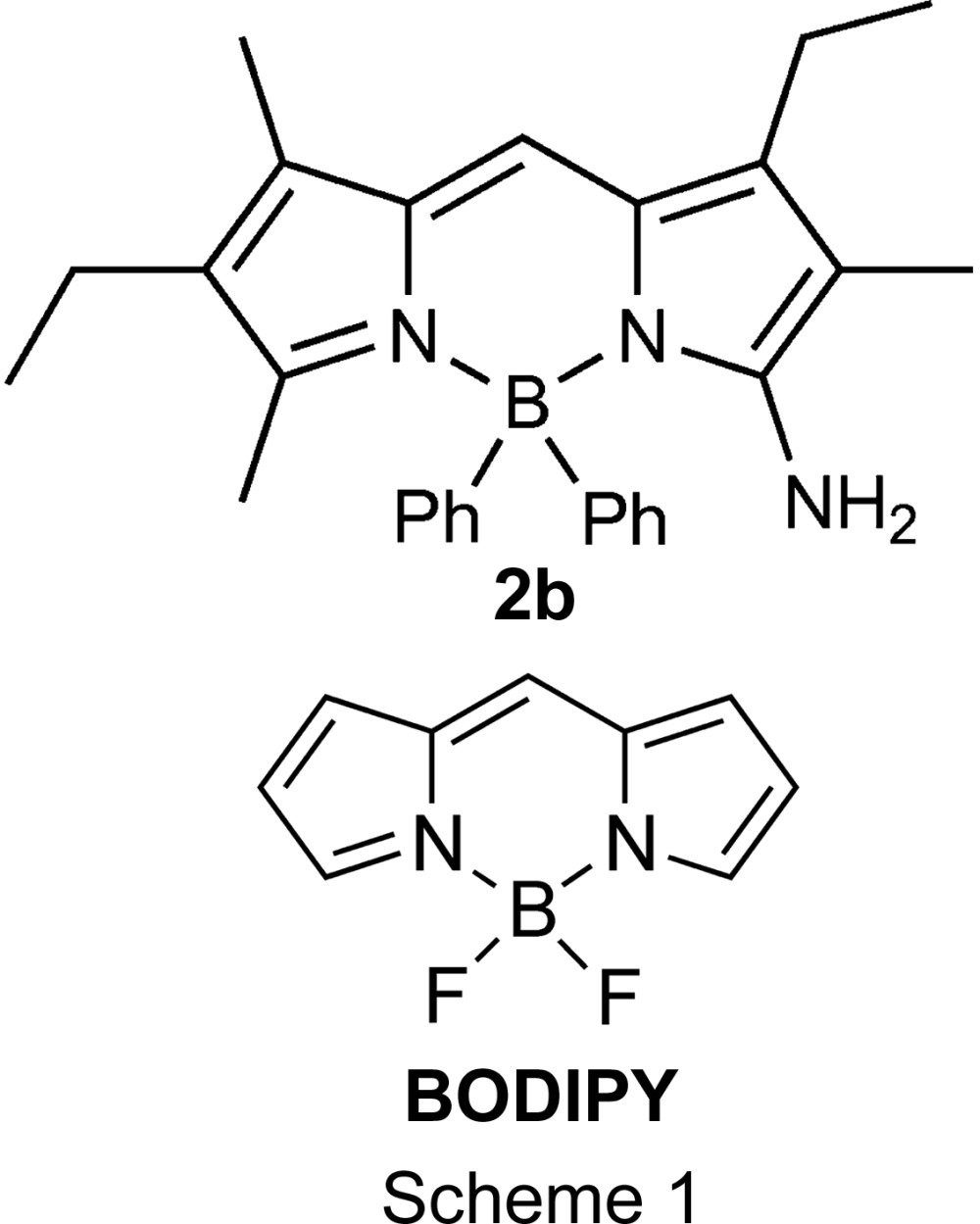


### Solvent-dependent behaviour of BODIPY 2b observed by NMR spectroscopy   

**Figure 2:**
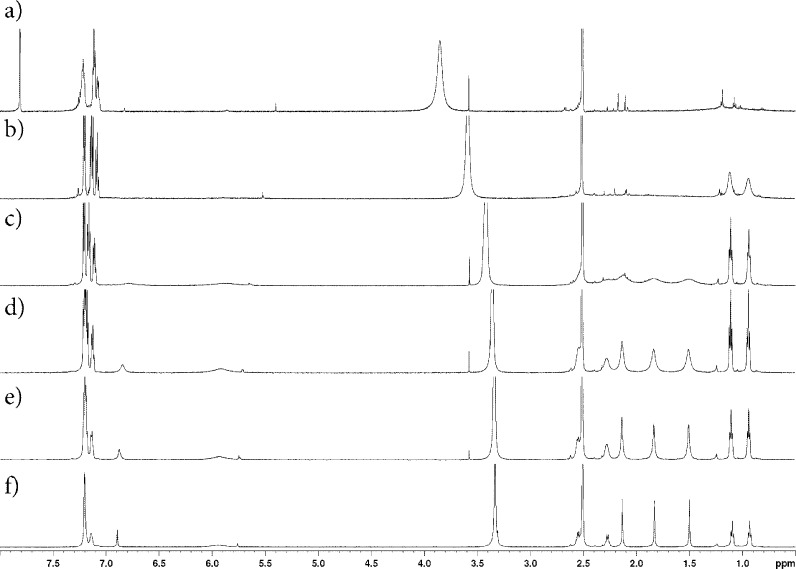
^1^H NMR spectra of BODIPY **2 b** in DMSO-*d*_6_ or mixtures of CDCl_3_ and DMSO-*d*_6_ in varying ratios: (*a*) DMSO-*d*_6_/CDCl_3_ (1:2 *v*/*v*); (*b*) DMSO-*d*_6_/CDCl_3_ (1:1 *v*/*v*); (*c*) DMSO-*d*_6_/CDCl_3_ (5:2 *v*/*v*); (*d*) DMSO-*d*_6_/CDCl_3_ (5:1 *v*/*v*); (*e*) DMSO-*d*_6_/CDCl_3_ (10:1 *v*/*v*); (*f*) neat DMSO-*d*_6_.

The characterization of **2b** by ^1^H NMR spectroscopy yielded intriguing results. While the proton signals in ^1^H NMR spectra are fully resolved in DMSO-*d*_6_ (as in Fig. 2 ▸ *f*), the aliphatic protons are completely coalesced in CDCl_3_. It is also observed that gradual addition of CDCl_3_ to a solution of **2b** in DMSO-*d*_6_ led to a loss of resolution of the aliphatic protons (Figs. 2 ▸ *b*–*e*).

In deuterated di­chloro­methane and 1,2-di­bromo­ethane, the ^1^H NMR spectra are similarly coalesced (data not shown). On the other hand, spectra are resolved in deuterated methanol and toluene (data not shown), despite the poor solubility of **2b** in methanol. These observations prompted us to further investigate the absorption and fluorescent emission behaviour of BODIPY **2b** in solution.

### Solvent-dependent behavior of BODIPY 2b observed by fluorescence spectroscopy   

**Figure 3:**
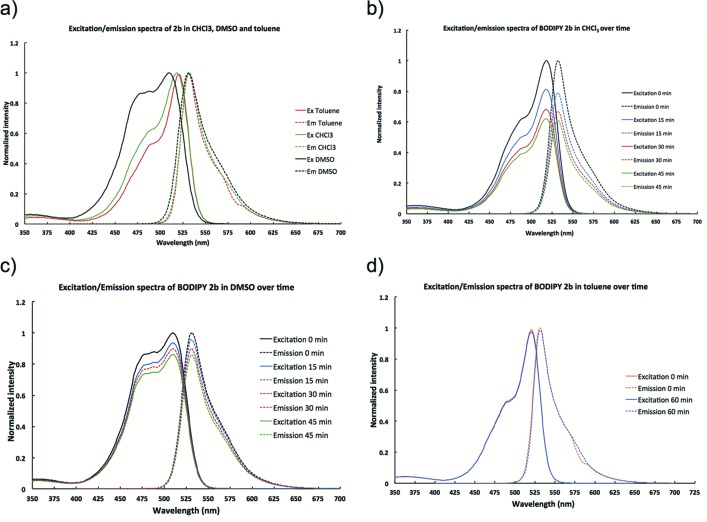
Excitation and emission profile of 3-amino-4,4-diphenyl-BODIPY **2 b** in (*a*) chloro­form, DMSO and toluene; (*b*) chloro­form over 45 min; (*c*) DMSO over 45 min; (*d*) toluene over 60 min.

Fig. 3 ▸(*a*) suggests that the fluorescence spectra of **2b** in chloro­form, and to some extend in DMSO as well, shows time-dependent fluorescent intensities. In contrast, most solvatochromic BODIPY fluoro­phores that have been reported in the literature often show different maximal emission wavelengths (Baruah *et al.*, 2006 ▸; Clemens *et al.*, 2008 ▸; Filarowski *et al.*, 2010 ▸, 2015 ▸; de Rezende *et al.*, 2014 ▸), however, those solvatochromic BODIPY dyes do not display a time-dependent change in fluorescent intensity.
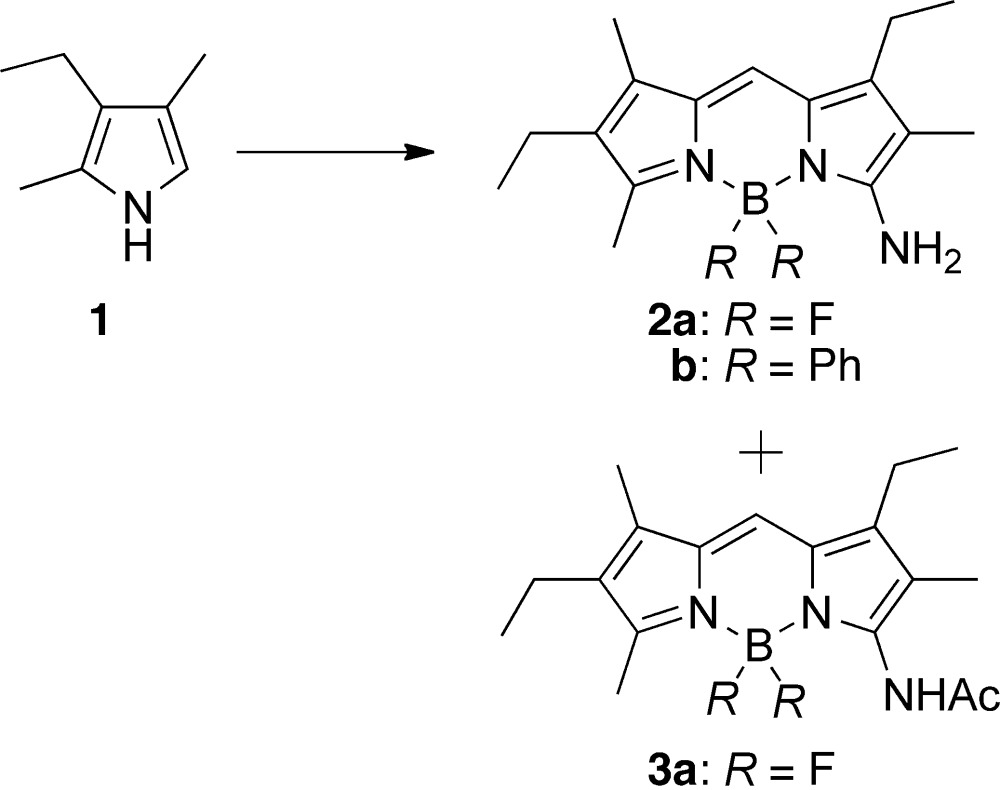


On the other hand, time-dependent spectroscopic changes, in emission intensity, shift of maximal emission wavelength, or absorbance, have been observed for compounds that undergo self-assembly in solution (Gassensmith *et al.*, 2007 ▸; Miyatake *et al.*, 2005 ▸). Taken together, these observations suggest that BODIPY **2b** shows a tendency to form assembled structures in chloro­form, not as significantly in DMSO, and particularly not in toluene.

**Figure 4:**
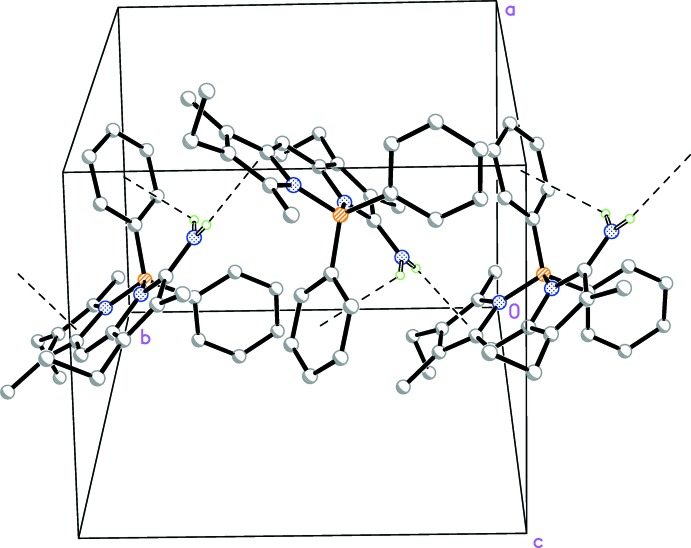
Part of the crystal structure of **2b**, with weak C—H⋯π inter­actions shown as dashed lines.

It can be seen from the crystal structure of BODIPY **2b** that the mol­ecules are linked along the BODIPY plane by inter­actions between one of the amino H atoms and the BODIPY π ring (N—H⋯π ring; Table 1 ▸ and Fig. 4 ▸).

It is conceivable that in solutions such as in di­chloro­methane, chloro­form and di­bromo­ethane, compound **2b** could maintain similar inter­molecular assemblies. As a consequence of the reduced mobility of the BODIPY mol­ecules in these assembled structures, the alkyl signals are broadened to the extent that they become invisible in the NMR spectra (Celis *et al.*, 2013 ▸; Brand *et al.*, 2008 ▸; Chen *et al.*, 2015 ▸). Motion of the phenyl rings, however, is not affected in the assembly, and thus the phenyl aromatic protons are visible in these solvents. In polar solvents such as DMSO and methanol, it is possible that solvation of the BODIPY NH_2_ group abolishes the ability for such assemblies to occur. On the other hand, in toluene, strong inter­actions of the aromatic benzene ring with the BODIPY co-plane could also diminish the assemblies. The emission profiles of BODIPY **2b** in DMSO, chloro­form and toluene also corroborate this model.

## Structural commentary   

The mol­ecular structure of **2b** shown in Fig. 1 ▸ displays a typical BODIPY structure (Tram *et al.*, 2009 ▸). The central six-membered ring has a flattened sofa conformation with atom N1 deviating by 0.142 (4) Å from the mean plane of the other five atoms (N2/C4/C5/C6/N1), which has an r.m.s. deviation of 0.015 Å. The dihedral angle between the two essentailly planar outer five-membered rings (N1/C1–C4 and N2/C6–C9) is 8.0 (2)°. The two B—N bond lengths are the same within experimental error [1.594 (4) and 1.579 (4) Å], confirming the delocalized nature of the BODIPY core. The two phenyl rings form dihedral angles of 78.8 (1) (C17–C22) and 80.8 (1)° (C23–C28) with the approximate plane of the 12 atoms of the BODIPY core (B1/N1/N2/C1–C9), which has an r.m.s. deviation of 0.067 Å. The dihedral angle between the two phenyl rings is 48.6 (2)°. Methyl atoms C12 and C15, belonging to the ethyl substituents, deviate by −1.326 (4) and 1.348 (3) Å, respectively, from the mean plane of the 12 atoms of the BODIPY core. There is a weak intra­molecular N3—H1N⋯π inter­action involving the amino group and the C17–C22 phenyl ring (Table 1 ▸).

## Supra­molecular features   

In the crystal, mol­ecules are linked *via* weak N—H⋯π inter­actions (Table 1 ▸), forming chains along [010] (Fig. 4 ▸).

## Spectroscopy and experimental   

Bruker Avance 300 and 600 Digital NMR spectrometers with a 14.1 and 7.05 Tesla Ultrashield magnet, respectively, were used to obtain ^1^H and ^11^B NMR spectra. ^1^H NMR spectra were measured at 300 or 600 MHz, and ^11^B at 96 MHz. Chemical shifts and coupling constants (*J* values) are given in ppm (δ) and Hz, respectively. Deuterated solvents were purchased from C/D/N Isotopes Inc. Fluorescence spectroscopy was recorded using a QuantaMaster model QM-2001-4 cuvette-based L-format scanning spectro­fluoro­meter from Photon Technology Inter­national (PTI), inter­faced with *FeliX32* software. UV–Vis spectra were obtained using a Thermospectronic/Unicam UV/Vis spectrometer configured to the *Vision32* software.

Anhydrous di­chloro­methane, tri­ethyl­amine and toluene were generated by first heating under reflux in the presence of phospho­rus pentoxide, calcium hydride and sodium metal, respectively, followed by distillation under an atmosphere of nitro­gen. All other chemicals and reagents were purchased from Sigma–Aldrich or TCI without further purification prior to use.

## Synthesis and crystallization   

For the preparation of **2b**, a solution of sodium nitrite (80 mg, 1.2 mmol) in water (1.0 ml) was added dropwise to another solution of 3-ethyl-2,4-di­methyl­pyrrole (0.25 ml, 1.85 mmol) in acetic acid (7.5 ml) and acetic anhydride (7.5 ml). The mixture was then heated at 373 K for 4 h. The solvents were removed under reduced pressure. The resulting products were diluted with di­chloro­methane (20 ml) and washed with a saturated aqueous sodium bicarbonate solution (2 × 15 ml). The organic phase was dried (MgSO_4_) and evaporated to dryness under reduced pressure. The residue was co-evaporated with dry toluene (10 ml) and then redissolved in dry di­chloro­methane (10 ml), followed by addition of dry tri­ethyl­amine (1.0 ml, 7.1 mmol). After stirring for 30 min, boron–di­phenyl­bromide (Noth & Vahrenkamp, 1968 ▸) (1.5 ml, 8.2 mmol) was added. Stirring was continued for 20 h and the products were washed with water (3 × 30 ml), dried (MgSO_4_) and evaporated under reduced pressure. The residue was purified by column chromatography on silica gel. The appropriate fractions, eluted with di­chloro­methane–hexane (1:9 *v*/*v*), were pooled and concentrated under reduced pressure to give the title compound as an orange solid (yield 18 mg, 4%). Single crystals were obtained by slow evaporation of the corresponding solution in hexane. δ_H_[DMSO-*d*_6_]: 7.19–7.64 (*br*, 10H), 6.89 (*s*, 1H), 5.94 (*br*, 2H), 2.55 (*q*, 2H, *J* = 7.5), 2.27 (*q*, 2H, *J* = 7.5 Hz), 2.13 (*s*, 3H), 1.83 (*s*, 3H), 1.50 (*s*, 3H), 1.09 (*t*, 2H, *J* = 7.5 Hz), 0.93 (*t*, 2H, *J* = 7.5 Hz). δ_B_[DMSO-*d*_6_]: 0.66 (*s*).

### Refinement   

Crystal data, data collection and structure refinement details are summarized in Table 2 ▸. H atoms bonded to C atoms were included in calculated positions, with C—H = 0.95–0.99 Å, and were allowed to refine in a riding-motion approximation, with *U*_iso_(H) = 1.2*U*_eq_(C) or 1.5*U*_eq_(C_meth­yl_). The amino H atoms were refined independently with isotropic displacement parameters.

## Supplementary Material

Crystal structure: contains datablock(s) I. DOI: 10.1107/S2056989017002213/sj5520sup1.cif

Structure factors: contains datablock(s) I. DOI: 10.1107/S2056989017002213/sj5520Isup2.hkl

CCDC reference: 1531986

Additional supporting information:  crystallographic information; 3D view; checkCIF report

## Figures and Tables

**Table 1:** Hydrogen-bond geometry (Å, °) *Cg*1 and *Cg*2 are the centroids of the C17–C22 and N2/C6–C9 rings, respectively.

*D*—H⋯*A*	*D*—H	H⋯*A*	*D*⋯*A*	*D*—H⋯*A*
N3—H1N⋯*Cg*1	0.87 (4)	3.07 (3)	3.772 (2)	139 (2)
N3—H2N⋯*Cg*2^i^	0.87 (4)	2.44 (3)	3.223 (2)	150 (2)

Symmetry code: (i) 
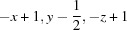
.

**Table 2:** Experimental details

Crystal data	
Chemical formula	C_28_H_32_BN_3_
*M*_r_	421.37
Crystal system, space group	Monoclinic, *P*2_1_
Temperature (K)	147
*a*, *b*, *c* (Å)	9.4938 (7), 11.5325 (8), 11.3739 (9)
β (°)	109.557 (2)
*V* (Å^3^)	1173.45 (15)
*Z*	2
Radiation type	Mo *K*α
μ (mm^−1^)	0.07
Crystal size (mm)	0.35 × 0.27 × 0.07

Data collection	
Diffractometer	Bruker Kappa APEX DUO CCD
Absorption correction	Multi-scan (*SADABS*; Bruker, 2014 ▸)
*T*_min_, *T*_max_	0.701, 0.746
No. of measured, independent and observed [*I* > 2σ(*I*)] reflections	10457, 5032, 4054
*R*_int_	0.040
(sin θ/λ)_max_ (Å^−1^)	0.650

Refinement	
*R*[*F* ^2^ > 2σ(*F* ^2^)], *wR*(*F* ^2^), *S*	0.046, 0.104, 1.03
No. of reflections	5032
No. of parameters	302
No. of restraints	1
H-atom treatment	H atoms treated by a mixture of independent and constrained refinement
Δρ_max_, Δρ_min_ (e Å^−3^)	0.19, −0.19
Absolute structure	Flack *x* determined using 1500 quotients [(*I* ^+^)−(*I* ^−^)]/[(*I* ^+^)+(*I* ^−^)] (Parsons *et al.*, 2013 ▸)
Absolute structure parameter	−1.3 (10)

Computer programs: *APEX2* and *SAINT* (Bruker, 2014 ▸), *SHELXT* (Sheldrick, 2015*a* ▸), *SHELXL2014* (Sheldrick, 2015*b* ▸), *PLATON* (Spek, 2009 ▸) and *SHELXTL* (Sheldrick, 2008 ▸).

## supplementary crystallographic information

### Crystal data

**Table:**

C_28_H_32_BN_3_	*F*(000) = 452
*M**_r_* = 421.37	*D*_x_ = 1.193 Mg m^−^^3^
Monoclinic, *P*2_1_	Mo *K*α radiation, λ = 0.71073 Å
*a* = 9.4938 (7) Å	Cell parameters from 4278 reflections
*b* = 11.5325 (8) Å	θ = 2.4–27.5°
*c* = 11.3739 (9) Å	µ = 0.07 mm^−^^1^
β = 109.557 (2)°	*T* = 147 K
*V* = 1173.45 (15) Å^3^	Plate, red
*Z* = 2	0.35 × 0.27 × 0.07 mm

### Data collection

**Table:**

Bruker Kappa APEX DUO CCD diffractometer	4054 reflections with *I* > 2σ(*I*)
Radiation source: sealed tube with Bruker Triumph monochromator	*R*_int_ = 0.040
φ and ω scans	θ_max_ = 27.5°, θ_min_ = 1.9°
Absorption correction: multi-scan (SADABS; Bruker, 2014)	*h* = −12→12
*T*_min_ = 0.701, *T*_max_ = 0.746	*k* = −14→11
10457 measured reflections	*l* = −14→14
5032 independent reflections	

### Refinement

**Table:**

Refinement on *F*^2^	Hydrogen site location: mixed
Least-squares matrix: full	H atoms treated by a mixture of independent and constrained refinement
*R*[*F*^2^ > 2σ(*F*^2^)] = 0.046	*w* = 1/[σ^2^(*F*_o_^2^) + (0.0464*P*)^2^ + 0.0459*P*] where *P* = (*F*_o_^2^ + 2*F*_c_^2^)/3
*wR*(*F*^2^) = 0.104	(Δ/σ)_max_ < 0.001
*S* = 1.03	Δρ_max_ = 0.19 e Å^−^^3^
5032 reflections	Δρ_min_ = −0.19 e Å^−^^3^
302 parameters	Absolute structure: Flack *x* determined using 1500 quotients [(I+)-(I-)]/[(I+)+(I-)] (Parsons *et al*, 2013)
1 restraint	Absolute structure parameter: −1.3 (10)

### Special details

**Table:**

Geometry. All e.s.d.'s (except the e.s.d. in the dihedral angle between two l.s. planes) are estimated using the full covariance matrix. The cell e.s.d.'s are taken into account individually in the estimation of e.s.d.'s in distances, angles and torsion angles; correlations between e.s.d.'s in cell parameters are only used when they are defined by crystal symmetry. An approximate (isotropic) treatment of cell e.s.d.'s is used for estimating e.s.d.'s involving l.s. planes.

### Fractional atomic coordinates and isotropic or equivalent isotropic displacement parameters (Å^2^)

**Table:**

	*x*	*y*	*z*	*U*_iso_*/*U*_eq_	
N1	0.6209 (2)	0.4011 (2)	0.47877 (19)	0.0171 (5)	
N2	0.7753 (2)	0.51637 (19)	0.66391 (19)	0.0174 (5)	
N3	0.4280 (3)	0.2624 (2)	0.4350 (3)	0.0291 (6)	
C1	0.5218 (3)	0.3302 (3)	0.4010 (3)	0.0204 (6)	
C2	0.5273 (3)	0.3383 (3)	0.2759 (3)	0.0223 (6)	
C3	0.6299 (3)	0.4212 (3)	0.2787 (2)	0.0191 (6)	
C4	0.6900 (3)	0.4631 (2)	0.4057 (2)	0.0174 (6)	
C5	0.7876 (3)	0.5506 (2)	0.4559 (2)	0.0187 (6)	
H5A	0.8298	0.5927	0.4041	0.022*	
C6	0.8277 (3)	0.5803 (2)	0.5833 (2)	0.0172 (6)	
C7	0.9196 (3)	0.6704 (2)	0.6497 (3)	0.0192 (6)	
C8	0.9259 (3)	0.6587 (2)	0.7743 (3)	0.0208 (6)	
C9	0.8385 (3)	0.5641 (3)	0.7804 (2)	0.0202 (6)	
C10	0.4334 (4)	0.2669 (3)	0.1690 (3)	0.0348 (8)	
H10A	0.4545	0.2885	0.0933	0.052*	
H10B	0.3275	0.2805	0.1566	0.052*	
H10C	0.4566	0.1846	0.1871	0.052*	
C11	0.6694 (3)	0.4692 (3)	0.1714 (3)	0.0243 (7)	
H11A	0.6559	0.4081	0.1075	0.029*	
H11B	0.7760	0.4924	0.2009	0.029*	
C12	0.5734 (4)	0.5736 (3)	0.1125 (3)	0.0434 (9)	
H12A	0.6030	0.6028	0.0433	0.065*	
H12B	0.5873	0.6348	0.1753	0.065*	
H12C	0.4680	0.5506	0.0811	0.065*	
C13	0.9925 (3)	0.7628 (3)	0.5969 (3)	0.0279 (7)	
H13A	1.0991	0.7672	0.6462	0.042*	
H13B	0.9451	0.8377	0.5998	0.042*	
H13C	0.9810	0.7439	0.5102	0.042*	
C14	1.0172 (3)	0.7322 (3)	0.8821 (3)	0.0260 (7)	
H14A	0.9688	0.7331	0.9469	0.031*	
H14B	1.0193	0.8129	0.8530	0.031*	
C15	1.1776 (3)	0.6883 (3)	0.9400 (3)	0.0365 (8)	
H15A	1.2328	0.7394	1.0089	0.055*	
H15B	1.2265	0.6880	0.8765	0.055*	
H15C	1.1765	0.6094	0.9716	0.055*	
C16	0.8189 (3)	0.5127 (3)	0.8948 (3)	0.0263 (7)	
H16A	0.7133	0.5161	0.8876	0.039*	
H16B	0.8787	0.5565	0.9684	0.039*	
H16C	0.8521	0.4317	0.9032	0.039*	
C17	0.5151 (3)	0.4325 (2)	0.6603 (2)	0.0182 (6)	
C18	0.4069 (3)	0.5069 (3)	0.5835 (3)	0.0269 (7)	
H18A	0.4245	0.5410	0.5136	0.032*	
C19	0.2748 (3)	0.5331 (3)	0.6051 (3)	0.0355 (8)	
H19A	0.2036	0.5835	0.5502	0.043*	
C20	0.2475 (3)	0.4855 (3)	0.7068 (3)	0.0347 (8)	
H20A	0.1579	0.5036	0.7228	0.042*	
C21	0.3512 (3)	0.4115 (3)	0.7851 (3)	0.0337 (8)	
H21A	0.3330	0.3783	0.8551	0.040*	
C22	0.4828 (3)	0.3855 (3)	0.7615 (3)	0.0261 (7)	
H22A	0.5528	0.3341	0.8161	0.031*	
C23	0.7586 (3)	0.2907 (2)	0.6849 (2)	0.0177 (6)	
C24	0.9149 (3)	0.2865 (3)	0.7172 (3)	0.0239 (6)	
H24A	0.9667	0.3550	0.7096	0.029*	
C25	0.9962 (3)	0.1863 (3)	0.7597 (3)	0.0301 (7)	
H25A	1.1018	0.1870	0.7805	0.036*	
C26	0.9244 (4)	0.0857 (3)	0.7719 (3)	0.0295 (7)	
H26A	0.9801	0.0169	0.8011	0.035*	
C27	0.7705 (4)	0.0853 (3)	0.7414 (3)	0.0276 (7)	
H27A	0.7201	0.0160	0.7491	0.033*	
C28	0.6900 (3)	0.1866 (3)	0.6996 (3)	0.0238 (6)	
H28A	0.5846	0.1853	0.6803	0.029*	
B1	0.6672 (3)	0.4083 (3)	0.6269 (3)	0.0181 (6)	
H2N	0.359 (4)	0.227 (3)	0.376 (3)	0.035 (10)*	
H1N	0.417 (4)	0.268 (3)	0.508 (4)	0.052 (12)*	

### Atomic displacement parameters (Å^2^)

**Table:**

	*U*^11^	*U*^22^	*U*^33^	*U*^12^	*U*^13^	*U*^23^
N1	0.0158 (11)	0.0172 (13)	0.0187 (11)	−0.0024 (9)	0.0062 (9)	0.0003 (9)
N2	0.0170 (11)	0.0171 (13)	0.0173 (11)	0.0007 (9)	0.0046 (9)	0.0019 (9)
N3	0.0315 (15)	0.0320 (17)	0.0225 (14)	−0.0164 (12)	0.0072 (12)	−0.0018 (12)
C1	0.0202 (14)	0.0194 (17)	0.0202 (14)	−0.0043 (11)	0.0048 (11)	−0.0022 (11)
C2	0.0221 (14)	0.0232 (18)	0.0201 (14)	−0.0028 (12)	0.0053 (11)	−0.0008 (12)
C3	0.0174 (13)	0.0207 (17)	0.0200 (13)	0.0019 (11)	0.0071 (11)	−0.0007 (11)
C4	0.0176 (13)	0.0166 (17)	0.0193 (14)	0.0011 (11)	0.0079 (11)	0.0030 (10)
C5	0.0168 (13)	0.0206 (16)	0.0203 (13)	0.0014 (11)	0.0084 (11)	0.0034 (11)
C6	0.0145 (13)	0.0170 (16)	0.0203 (13)	0.0004 (10)	0.0057 (10)	0.0016 (11)
C7	0.0157 (13)	0.0168 (17)	0.0240 (14)	0.0000 (11)	0.0052 (11)	−0.0002 (11)
C8	0.0189 (13)	0.0185 (18)	0.0232 (15)	0.0019 (11)	0.0046 (11)	−0.0023 (12)
C9	0.0168 (13)	0.0221 (17)	0.0195 (14)	0.0029 (11)	0.0031 (11)	−0.0008 (11)
C10	0.042 (2)	0.034 (2)	0.0282 (16)	−0.0144 (15)	0.0110 (14)	−0.0074 (15)
C11	0.0289 (15)	0.0261 (18)	0.0208 (15)	−0.0028 (13)	0.0120 (12)	−0.0013 (11)
C12	0.055 (2)	0.044 (2)	0.0372 (19)	0.0168 (17)	0.0241 (17)	0.0187 (17)
C13	0.0289 (16)	0.0236 (18)	0.0307 (16)	−0.0063 (13)	0.0095 (13)	−0.0004 (13)
C14	0.0299 (17)	0.0221 (18)	0.0247 (15)	−0.0037 (12)	0.0072 (13)	−0.0064 (12)
C15	0.0292 (17)	0.040 (2)	0.0307 (18)	−0.0052 (15)	−0.0023 (14)	−0.0083 (14)
C16	0.0303 (15)	0.0278 (18)	0.0209 (15)	−0.0040 (13)	0.0086 (12)	−0.0006 (12)
C17	0.0170 (13)	0.0158 (16)	0.0217 (14)	−0.0020 (10)	0.0062 (10)	−0.0024 (11)
C18	0.0237 (15)	0.0267 (18)	0.0306 (16)	0.0032 (12)	0.0094 (12)	0.0051 (13)
C19	0.0257 (16)	0.035 (2)	0.0425 (19)	0.0076 (14)	0.0074 (14)	0.0016 (15)
C20	0.0220 (15)	0.035 (2)	0.052 (2)	−0.0011 (13)	0.0191 (15)	−0.0099 (15)
C21	0.0334 (18)	0.040 (2)	0.0364 (18)	−0.0022 (15)	0.0229 (15)	0.0010 (15)
C22	0.0236 (15)	0.0269 (19)	0.0281 (16)	0.0017 (12)	0.0090 (12)	0.0027 (12)
C23	0.0215 (14)	0.0186 (16)	0.0144 (13)	−0.0013 (11)	0.0079 (11)	−0.0025 (10)
C24	0.0230 (15)	0.0221 (17)	0.0279 (15)	−0.0006 (12)	0.0103 (12)	−0.0002 (12)
C25	0.0235 (15)	0.0296 (19)	0.0366 (18)	0.0065 (13)	0.0094 (13)	−0.0004 (14)
C26	0.0336 (18)	0.0218 (19)	0.0304 (17)	0.0104 (13)	0.0071 (13)	0.0037 (13)
C27	0.0345 (17)	0.0174 (18)	0.0305 (16)	0.0002 (12)	0.0103 (13)	0.0019 (12)
C28	0.0206 (14)	0.0246 (18)	0.0249 (15)	−0.0028 (12)	0.0057 (12)	0.0019 (12)
B1	0.0190 (15)	0.0184 (18)	0.0170 (15)	−0.0018 (12)	0.0060 (12)	0.0017 (12)

### Geometric parameters (Å, º)

**Table:**

N1—C1	1.334 (3)	C13—H13C	0.9800
N1—C4	1.413 (3)	C14—C15	1.529 (4)
N1—B1	1.594 (4)	C14—H14A	0.9900
N2—C9	1.374 (3)	C14—H14B	0.9900
N2—C6	1.392 (3)	C15—H15A	0.9800
N2—B1	1.579 (4)	C15—H15B	0.9800
N3—C1	1.335 (4)	C15—H15C	0.9800
N3—H2N	0.87 (4)	C16—H16A	0.9800
N3—H1N	0.87 (4)	C16—H16B	0.9800
C1—C2	1.445 (4)	C16—H16C	0.9800
C2—C3	1.358 (4)	C17—C22	1.396 (4)
C2—C10	1.492 (4)	C17—C18	1.398 (4)
C3—C4	1.446 (4)	C17—B1	1.635 (4)
C3—C11	1.498 (4)	C18—C19	1.389 (4)
C4—C5	1.360 (4)	C18—H18A	0.9500
C5—C6	1.411 (4)	C19—C20	1.382 (5)
C5—H5A	0.9500	C19—H19A	0.9500
C6—C7	1.404 (4)	C20—C21	1.379 (5)
C7—C8	1.405 (4)	C20—H20A	0.9500
C7—C13	1.501 (4)	C21—C22	1.396 (4)
C8—C9	1.386 (4)	C21—H21A	0.9500
C8—C14	1.505 (4)	C22—H22A	0.9500
C9—C16	1.496 (4)	C23—C28	1.403 (4)
C10—H10A	0.9800	C23—C24	1.405 (4)
C10—H10B	0.9800	C23—B1	1.626 (4)
C10—H10C	0.9800	C24—C25	1.383 (4)
C11—C12	1.523 (4)	C24—H24A	0.9500
C11—H11A	0.9900	C25—C26	1.377 (5)
C11—H11B	0.9900	C25—H25A	0.9500
C12—H12A	0.9800	C26—C27	1.384 (4)
C12—H12B	0.9800	C26—H26A	0.9500
C12—H12C	0.9800	C27—C28	1.391 (4)
C13—H13A	0.9800	C27—H27A	0.9500
C13—H13B	0.9800	C28—H28A	0.9500
			
C1—N1—C4	106.5 (2)	C8—C14—C15	112.5 (3)
C1—N1—B1	128.0 (2)	C8—C14—H14A	109.1
C4—N1—B1	125.2 (2)	C15—C14—H14A	109.1
C9—N2—C6	106.6 (2)	C8—C14—H14B	109.1
C9—N2—B1	127.5 (2)	C15—C14—H14B	109.1
C6—N2—B1	125.9 (2)	H14A—C14—H14B	107.8
C1—N3—H2N	117 (2)	C14—C15—H15A	109.5
C1—N3—H1N	122 (3)	C14—C15—H15B	109.5
H2N—N3—H1N	118 (3)	H15A—C15—H15B	109.5
N1—C1—N3	123.9 (3)	C14—C15—H15C	109.5
N1—C1—C2	111.3 (2)	H15A—C15—H15C	109.5
N3—C1—C2	124.8 (3)	H15B—C15—H15C	109.5
C3—C2—C1	106.5 (2)	C9—C16—H16A	109.5
C3—C2—C10	129.6 (3)	C9—C16—H16B	109.5
C1—C2—C10	123.9 (3)	H16A—C16—H16B	109.5
C2—C3—C4	107.4 (2)	C9—C16—H16C	109.5
C2—C3—C11	127.9 (3)	H16A—C16—H16C	109.5
C4—C3—C11	124.6 (3)	H16B—C16—H16C	109.5
C5—C4—N1	120.9 (2)	C22—C17—C18	115.8 (3)
C5—C4—C3	130.7 (2)	C22—C17—B1	125.5 (2)
N1—C4—C3	108.3 (2)	C18—C17—B1	118.7 (2)
C4—C5—C6	121.6 (3)	C19—C18—C17	122.7 (3)
C4—C5—H5A	119.2	C19—C18—H18A	118.6
C6—C5—H5A	119.2	C17—C18—H18A	118.6
N2—C6—C7	109.5 (2)	C20—C19—C18	119.7 (3)
N2—C6—C5	120.9 (2)	C20—C19—H19A	120.2
C7—C6—C5	129.6 (3)	C18—C19—H19A	120.2
C6—C7—C8	106.2 (2)	C21—C20—C19	119.6 (3)
C6—C7—C13	126.7 (2)	C21—C20—H20A	120.2
C8—C7—C13	127.1 (2)	C19—C20—H20A	120.2
C9—C8—C7	107.6 (2)	C20—C21—C22	120.0 (3)
C9—C8—C14	126.4 (3)	C20—C21—H21A	120.0
C7—C8—C14	125.9 (3)	C22—C21—H21A	120.0
N2—C9—C8	110.0 (2)	C17—C22—C21	122.2 (3)
N2—C9—C16	122.6 (3)	C17—C22—H22A	118.9
C8—C9—C16	127.3 (2)	C21—C22—H22A	118.9
C2—C10—H10A	109.5	C28—C23—C24	115.5 (3)
C2—C10—H10B	109.5	C28—C23—B1	123.8 (2)
H10A—C10—H10B	109.5	C24—C23—B1	120.6 (2)
C2—C10—H10C	109.5	C25—C24—C23	122.5 (3)
H10A—C10—H10C	109.5	C25—C24—H24A	118.7
H10B—C10—H10C	109.5	C23—C24—H24A	118.7
C3—C11—C12	112.0 (3)	C26—C25—C24	120.1 (3)
C3—C11—H11A	109.2	C26—C25—H25A	120.0
C12—C11—H11A	109.2	C24—C25—H25A	120.0
C3—C11—H11B	109.2	C25—C26—C27	119.7 (3)
C12—C11—H11B	109.2	C25—C26—H26A	120.2
H11A—C11—H11B	107.9	C27—C26—H26A	120.2
C11—C12—H12A	109.5	C26—C27—C28	119.7 (3)
C11—C12—H12B	109.5	C26—C27—H27A	120.1
H12A—C12—H12B	109.5	C28—C27—H27A	120.1
C11—C12—H12C	109.5	C27—C28—C23	122.4 (3)
H12A—C12—H12C	109.5	C27—C28—H28A	118.8
H12B—C12—H12C	109.5	C23—C28—H28A	118.8
C7—C13—H13A	109.5	N2—B1—N1	104.3 (2)
C7—C13—H13B	109.5	N2—B1—C23	109.8 (2)
H13A—C13—H13B	109.5	N1—B1—C23	107.8 (2)
C7—C13—H13C	109.5	N2—B1—C17	110.4 (2)
H13A—C13—H13C	109.5	N1—B1—C17	107.6 (2)
H13B—C13—H13C	109.5	C23—B1—C17	116.1 (2)
			
C4—N1—C1—N3	−175.8 (3)	C2—C3—C11—C12	89.8 (4)
B1—N1—C1—N3	9.8 (5)	C4—C3—C11—C12	−85.5 (4)
C4—N1—C1—C2	3.0 (3)	C9—C8—C14—C15	91.3 (4)
B1—N1—C1—C2	−171.4 (2)	C7—C8—C14—C15	−85.5 (4)
N1—C1—C2—C3	−2.6 (3)	C22—C17—C18—C19	−0.2 (5)
N3—C1—C2—C3	176.2 (3)	B1—C17—C18—C19	−179.6 (3)
N1—C1—C2—C10	177.9 (3)	C17—C18—C19—C20	0.6 (5)
N3—C1—C2—C10	−3.3 (5)	C18—C19—C20—C21	−0.6 (5)
C1—C2—C3—C4	1.0 (3)	C19—C20—C21—C22	0.2 (5)
C10—C2—C3—C4	−179.5 (3)	C18—C17—C22—C21	−0.3 (4)
C1—C2—C3—C11	−175.0 (3)	B1—C17—C22—C21	179.0 (3)
C10—C2—C3—C11	4.5 (5)	C20—C21—C22—C17	0.3 (5)
C1—N1—C4—C5	173.9 (3)	C28—C23—C24—C25	0.6 (4)
B1—N1—C4—C5	−11.4 (4)	B1—C23—C24—C25	−176.0 (3)
C1—N1—C4—C3	−2.3 (3)	C23—C24—C25—C26	−0.1 (5)
B1—N1—C4—C3	172.3 (2)	C24—C25—C26—C27	0.0 (5)
C2—C3—C4—C5	−175.0 (3)	C25—C26—C27—C28	−0.4 (5)
C11—C3—C4—C5	1.1 (5)	C26—C27—C28—C23	1.0 (4)
C2—C3—C4—N1	0.8 (3)	C24—C23—C28—C27	−1.0 (4)
C11—C3—C4—N1	176.9 (2)	B1—C23—C28—C27	175.4 (3)
N1—C4—C5—C6	2.2 (4)	C9—N2—B1—N1	175.5 (2)
C3—C4—C5—C6	177.5 (3)	C6—N2—B1—N1	−6.1 (3)
C9—N2—C6—C7	−2.0 (3)	C9—N2—B1—C23	−69.2 (3)
B1—N2—C6—C7	179.3 (2)	C6—N2—B1—C23	109.2 (3)
C9—N2—C6—C5	177.7 (2)	C9—N2—B1—C17	60.1 (3)
B1—N2—C6—C5	−1.0 (4)	C6—N2—B1—C17	−121.5 (3)
C4—C5—C6—N2	3.9 (4)	C1—N1—B1—N2	−174.3 (3)
C4—C5—C6—C7	−176.5 (3)	C4—N1—B1—N2	12.3 (3)
N2—C6—C7—C8	1.5 (3)	C1—N1—B1—C23	69.0 (3)
C5—C6—C7—C8	−178.1 (3)	C4—N1—B1—C23	−104.5 (3)
N2—C6—C7—C13	−176.8 (3)	C1—N1—B1—C17	−57.0 (4)
C5—C6—C7—C13	3.5 (5)	C4—N1—B1—C17	129.6 (3)
C6—C7—C8—C9	−0.4 (3)	C28—C23—B1—N2	161.5 (2)
C13—C7—C8—C9	177.9 (3)	C24—C23—B1—N2	−22.2 (3)
C6—C7—C8—C14	176.9 (3)	C28—C23—B1—N1	−85.4 (3)
C13—C7—C8—C14	−4.8 (4)	C24—C23—B1—N1	90.9 (3)
C6—N2—C9—C8	1.8 (3)	C28—C23—B1—C17	35.3 (4)
B1—N2—C9—C8	−179.6 (2)	C24—C23—B1—C17	−148.4 (2)
C6—N2—C9—C16	−174.8 (3)	C22—C17—B1—N2	−104.1 (3)
B1—N2—C9—C16	3.8 (4)	C18—C17—B1—N2	75.2 (3)
C7—C8—C9—N2	−0.9 (3)	C22—C17—B1—N1	142.6 (3)
C14—C8—C9—N2	−178.1 (3)	C18—C17—B1—N1	−38.1 (3)
C7—C8—C9—C16	175.5 (3)	C22—C17—B1—C23	21.8 (4)
C14—C8—C9—C16	−1.7 (5)	C18—C17—B1—C23	−158.9 (3)

### Hydrogen-bond geometry (Å, º)

Cg1 and Cg2 are the centroids of the C17-C22 and N2/C6-C9 rings, respectively.

**Table:**

*D*—H···*A*	*D*—H	H···*A*	*D*···*A*	*D*—H···*A*
N3—H1*N*···*Cg*1	0.87 (4)	3.07 (3)	3.772 (2)	139 (2)
N3—H2*N*···*Cg*2^i^	0.87 (4)	2.44 (3)	3.223 (2)	150 (2)

Symmetry code: (i) −*x*+1, *y*−1/2, −*z*+1.
